# The long road to elimination: malaria mortality in a South African population cohort over 21 years

**DOI:** 10.1017/gheg.2017.7

**Published:** 2017-07-25

**Authors:** P. Byass, M. A. Collinson, C. Kabudula, F. X. Gómez-Olivé, R. G. Wagner, S. Ngobeni, B. Silaule, P. Mee, M. Coetzee, W. Twine, S. M. Tollman, K. Kahn

**Affiliations:** 1 MRC-Wits Rural Public Health and Health Transitions Research Unit (Agincourt), School of Public Health, Faculty of Health Sciences, University of the Witwatersrand, Johannesburg, South Africa; 2 Division of Epidemiology and Global Health, Department of Public Health and Clinical Medicine, Umeå Centre for Global Health Research, Umeå University, Umeå, Sweden; 3 INDEPTH Network, Accra, Ghana; 4 Department of Global Health and Development, Faculty of Public Health and Policy, London School of Hygiene and Tropical Medicine, London, UK; 5 Faculty of Health Sciences, Wits Research Institute for Malaria, University of the Witwatersrand, Johannesburg, South Africa; 6 School of Animal, Plant and Environmental Sciences, University of the Witwatersrand, Johannesburg, South Africa

**Keywords:** Climate, disease control, malaria elimination, migration, South Africa

## Abstract

**Background:**

Malaria elimination is on global agendas following successful transmission reductions. Nevertheless moving from low to zero transmission is challenging. South Africa has an elimination target of 2018, which may or may not be realised in its hypoendemic areas.

**Methods:**

The Agincourt Health and Demographic Surveillance System has monitored population health in north-eastern South Africa since 1992. Malaria deaths were analysed against individual factors, socioeconomic status, labour migration and weather over a 21-year period, eliciting trends over time and associations with covariates.

**Results:**

Of 13 251 registered deaths over 1.58 million person-years, 1.2% were attributed to malaria. Malaria mortality rates increased from 1992 to 2013, while mean daily maximum temperature rose by 1.5 °C. Travel to endemic Mozambique became easier, and malaria mortality increased in higher socioeconomic groups. Overall, malaria mortality was significantly associated with age, socioeconomic status, labour migration and employment, yearly rainfall and higher rainfall/temperature shortly before death.

**Conclusions:**

Malaria persists as a small but important cause of death in this semi-rural South African population. Detailed longitudinal population data were crucial for these analyses. The findings highlight practical political, socioeconomic and environmental difficulties that may also be encountered elsewhere in moving from low-transmission scenarios to malaria elimination.

## Background

Various agendas towards malaria elimination (interruption of transmission within a defined area) have been postulated over the last 60 years, but with limited success. The recently concluded Millennium Development Goal 6 aimed for declining malaria incidence by 2015, which was achieved at the global level, and the new Sustainable Development Goal 3 aims to end epidemic malaria by 2030. Thus, in the light of encouraging progress by malaria control programmes in various settings, malaria elimination is back on the global agenda [1]. As with any disease elimination scenario, the process becomes more difficult as cases become rarer. Given the complexity of the human–mosquito malaria life cycle, variations in population immunity and the role of asymptomatic or chronic infection, moving from very low levels of malaria transmission to elimination is not easy [2]. Perhaps as a consequence, in the context of relatively very low transmission in Southern Africa, the World Health Organization's (WHO) 2015 World Malaria Report notes recent increases rather than decreases in numbers of cases in Botswana, Namibia and South Africa [3]. Much population-based malaria research rightly concentrates on higher-transmission settings, particularly in Africa, and consequently population-based understandings of hypoendemic malaria around the fringes of transmission zones are less well developed [4]. Additionally, insecticide resistance among mosquitoes is an increasing problem in many locations [5], and the susceptibility of malaria transmission to climate change is an emerging consideration [6].

South Africa is largely malaria-free, but north-eastern regions bordering Mozambique and Zimbabwe, which also lie at lower altitudes (typically from sea level to about 500 m, the so-called *lowveld*) are known areas of seasonal hypoendemic transmission. Malaria is recognised as a public health problem, particularly during the hotter and wetter summer season. The main malaria vectors in the region are *Anopheles arabiensis* and *Anopheles funestus* [7]. There is a current national agenda for moving towards elimination by 2018, which may or may not be achievable [8]. A report using routinely collected health facility data from the Limpopo Province between 1998 and 2007 found that malaria incidence was 1.2 per 1000 (decreasing from 1.7 to 0.5 over the period), with a case-fatality rate of 1.1%. For Limpopo's south-eastern District of Bohlabela, which borders the Agincourt population studied here, the malaria mortality rate for 1998–2006 was estimated at 0.02 per 1000. [9] Similar data from the adjoining Mpumalanga Province between 2002 and 2012 found a rather stable incidence rate of 57 per 1000 specifically in Bushbuckridge Municipality (the northern part of which includes the Agincourt population studied in this paper), which, combined with the Province-wide case-fatality rate of 0.63%, corresponded to a malaria mortality rate of 0.36 per 1000 [10]. The South African National Parks authority, responsible for the Kruger National Park which adjoins the Agincourt sub-district and provides substantial employment for Agincourt residents, specifically warns visitors of the risks of contracting malaria in this region of South Africa [11]. Cross-border regional initiatives to control malaria transmission and move towards elimination in the wider region included the Lubombo Spatial Development Initiative (LSDI) from 1999 to 2011. After LSDI ended in 2011, a resurgence in malaria burden led to the MOSASWA malaria initiative between Mozambique, South Africa and Swaziland being launched in 2015 [12].

**Fig. 1.**
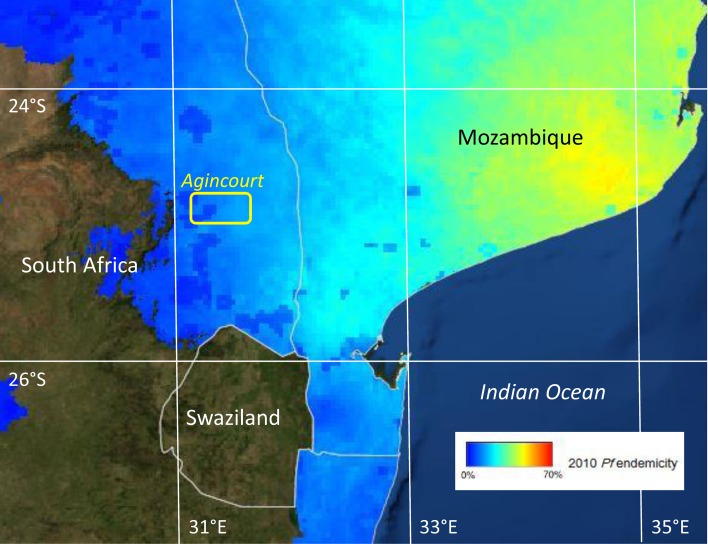
*Plasmodium falciparum* endemicity in southern Africa, showing the location of the Agincourt HDSS field site within the *P. falciparum* endemic area. (*P. falciparum* endemicity data sourced from http://www.map.ox.ac.uk/explorer/)

This paper focuses on long-term population-based data on malaria mortality from a Health and Demographic Surveillance System (HDSS) in the Agincourt sub-district, in north-eastern South Africa, to understand how epidemiological, social and climatic factors may have influenced continuing sporadic transmission from 1992 to 2013 [13]. The location of the Agincourt site, just south of the Tropic of Capricorn, is shown against modelled estimates of *Plasmodium falciparum* endemicity in Fig. 1. Using consistently documented mortality data and background information, the aim of this paper is to characterise the details of malaria mortality in the Agincourt area against a range of potential determinants. These findings are used as a basis for discussing the challenges of moving towards malaria elimination in South Africa and other low-transmission settings.

## Methods

The Agincourt HDSS was established in Bushbuckridge District, Mpumalanga Province, during 1992 as a means of tracking population-based health and disease in a rural South African population, formerly designated as a black homeland [13]. The HDSS area lies within South Africa's designated malaria endemic zone in north-eastern Mpumalanga, on the borders of Kruger National Park. All households in the designated area were initially documented and thereafter followed up annually. At the annual follow-up visits, any deaths that had occurred were investigated by means of a verbal autopsy (VA) interview. The Agincourt HDSS was a founding member of the INDEPTH Network, an umbrella organisation for many such population surveillance sites in Africa and Asia [14].

The INDEPTH Network has reported malaria mortality findings relating to more than 6000 malaria deaths across many of its constituent sites, and the methods used here are the same as those in that wider study [15]. These methods have been shown to have high co-validity with Global Burden of Disease cause-specific mortality estimates, across a wide range of low- and middle-income countries, including for malaria [16]. VA interviews are conducted by a trained field worker according to the structured format. At the start of the Agincourt surveillance there were no widely adopted standard structures for VA interviews, though standards have developed over the years, led largely by WHO and including input from Agincourt [17]. More recently, standardised automatic interpretation of VA interview material has become possible, including retrospectively, using computer models [18, 19]. The InterVA-4 model (version 4.02) has been used here to retrospectively process the Agincourt VA data consistently for the whole period 1992–2013, thus eliminating any case-by-case subjectivity or inter-observer variation [20]. InterVA-4 produces cause(s) of death with associated likelihoods for each case, and in these analyses all cases where the likelihood of malaria exceeded 50% were included. The distribution of likelihood among the malaria cases was skewed towards 100%, with a median likelihood of 85%. The ‘low’ malaria setting was used for the InterVA-4 model throughout to reflect the known low levels of malaria mortality in South African endemic areas [15].

Basic population data including individual at-risk exposure time (residence in the defined study area) and other relevant population-based variables were extracted from the Agincourt database for the 21-year period 1 September 1992 to 31 August 2013. Since malaria transmission in this area tends to be higher during the southern summer, around the turn of the year, the overall time period was divided into 21 one-year periods starting on 1st September each year, rather than using calendar years. Data items included age at death, date of death, household location, socioeconomic status of the household (measured in 2001, 2003, 2005, 2007, 2009 and 2013, as described and used previously [21]), settled in Agincourt but originating (generally via earlier generations of refugees) from Mozambique [22], and being either permanently resident or periodically away as a labour migrant in a particular year. The Agincourt definition of a labour migrant is a person who has a household to which they belong in a surveyed village, but who has been absent for more than half of the 12 months preceding interview. With many employment opportunities based some distance away, the majority of labour migrants are absent from Mpumalanga Province for long periods, largely residing and working in non-endemic areas of South Africa. Socioeconomic quartiles were constructed separately for each year to give a time-independent measure of relative socioeconomic status. Following the change of government in 1994, travel between South Africa and Mozambique became easier, with visa requirements for travel being finally dropped in 2005, though no individual data on travel to Mozambique were available [23]. Anecdotally, however, short-term visits by Agincourt residents to Mozambique for social and commercial purposes do occur. Given the lack of individual data on this, however, we were only able to compare overall malaria mortality in the periods before and after the visa requirements changed. Some routine vector control measures are also understood to have been undertaken in the Agincourt area at points during the overall period, but no locally detailed data were available on these activities.

Weather data on a daily basis for the same 21-year period and specifically for the Agincourt area were sourced from the European Centre for Medium-Range Weather Forecasts using the ERA-Interim model [24]. Daily rainfall, maximum temperature and minimum temperature data were used to calculate monthly and yearly averages. Following a pilot study of weather measurement in the Agincourt site in 2010 [25], it was possible to validate ERA-Interim daily maximum and minimum temperatures against observations for that 9-day period. ERA-Interim daily maximum temperatures ranged from 24.9 to 32.4 °C and minimum 18.5–21.7 °C, compared with observations of daily maximum temperature from 24.8 to 34.0 °C and minimum 18.8–22.8 °C. The mean difference in daily maximum was 1.4 °C and in minimum 0.7 °C. Since there are many ways in which weather parameters might relate to malaria transmission, not necessarily implying linear relationships between weather and mortality [26], quartile ranges of variables were used to avoid imposing mathematical assumptions on possible associations. Yearly means of daily maximum temperature and total rainfall were used to examine long-term influences, and monthly values for the month preceding malaria deaths used to examine short-term influences. Since both age group and chronological time were included in the data, survival regression models could not be used, and accordingly Poisson regression models were constructed to examine associations between malaria mortality rate ratios and possible epidemiological, social and climatic determinants. Including weather data aggregated on a monthly basis required a model separately characterising covariates for each person-month of exposure, leading to over 22 million observation points. Stata 12 software was used for these analyses. Spatiotemporal analysis for household clustering of malaria deaths was carried out using SaTScan 9.4.1.

## Results

A total of 165 malaria deaths were documented over 1.58 million person-years observed during a 21-year period from 1 September 1992 to 31 August 2013. This corresponded to a crude malaria mortality rate of 0.10 per 1000 person-years. All-cause deaths in the same population numbered 13 251, and hence malaria accounted for 1.2% of all mortality. Established diagnoses of malaria were reported as part of VA narratives for 45/165 cases (27.3%) and 62 cases (37.6%) were reported to have received unspecified intravenous treatment before death. No more detailed clinical information was available, although it was clear from the VA narratives that a proportion of the cases died without medical intervention, and probably would not therefore have been represented in facility-based statistics.

**Fig. 2.**
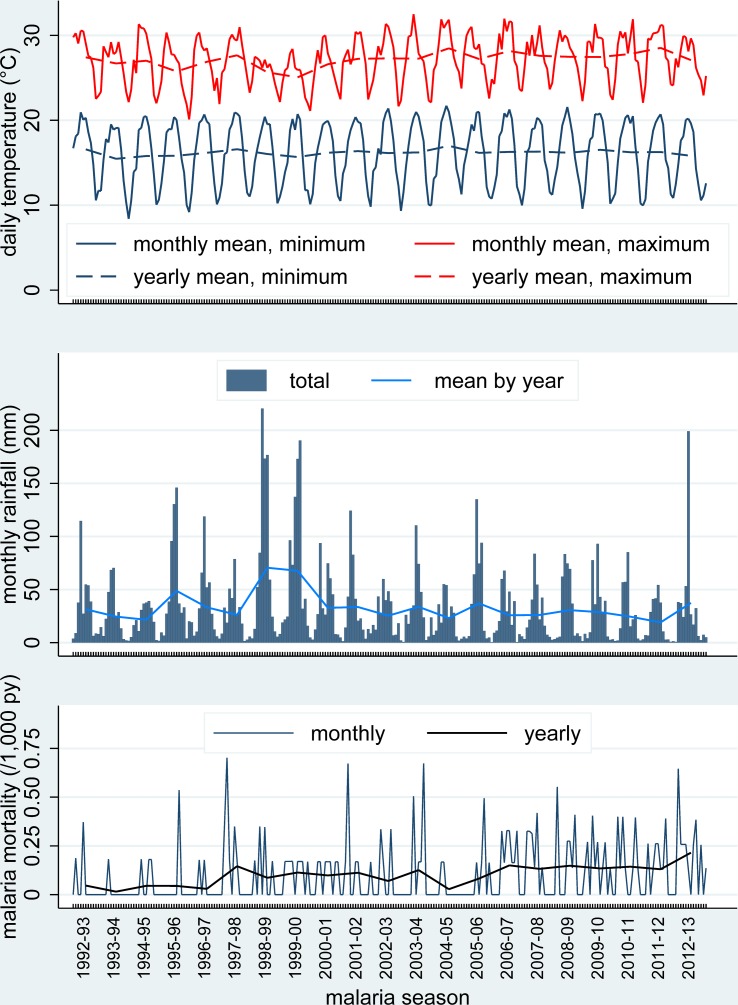
Mean maximum and minimum daily temperatures (monthly and annual means), rainfall [monthly totals (bars) and monthly means for each year] and malaria mortality rates (monthly and yearly values) for 165 malaria deaths over 1.58 million person-years at the Agincourt HDSS, South Africa, from 1992 to 2012.

**Fig. 3.**
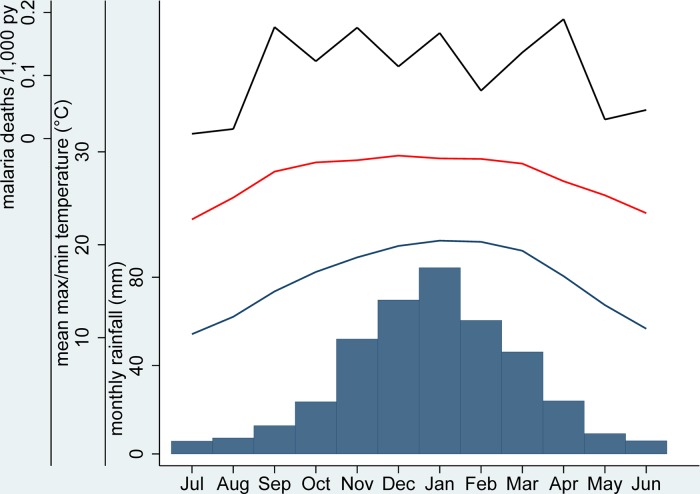
Maximum (red line) and minimum (blue line) daily mean temperatures, monthly rainfall (bars) and malaria mortality rates (black line), aggregated by calendar month for 165 malaria deaths over 1.58 million person-years at the Agincourt HDSS, South Africa, from 1992 to 2012.

Figure 2 shows malaria mortality in relation to temperature and weather data. Yearly means of maximum daily temperature rose steadily over the period, with an overall increase of 1.5 °C (95% CI 0.29–2.7). There were no significant trends in yearly means of minimum daily temperature or total yearly rainfall over the period, although the latter varied widely from year to year. Malaria mortality also varied considerably from month to month and year to year. It was higher in the later part of the overall period, when mean daily temperatures were higher and after visa requirements for travel between South Africa and Mozambique were lifted in 2005. Figure 3 shows the same parameters as in Fig. 2, but is presented as aggregate values over the 21 malaria seasons represented. An analysis of possible spatiotemporal clustering of malaria deaths by household was carried out, but none of the clusters identified by the SaTScan software had mortality rates significantly higher than the general population.

**Fig. 4.**
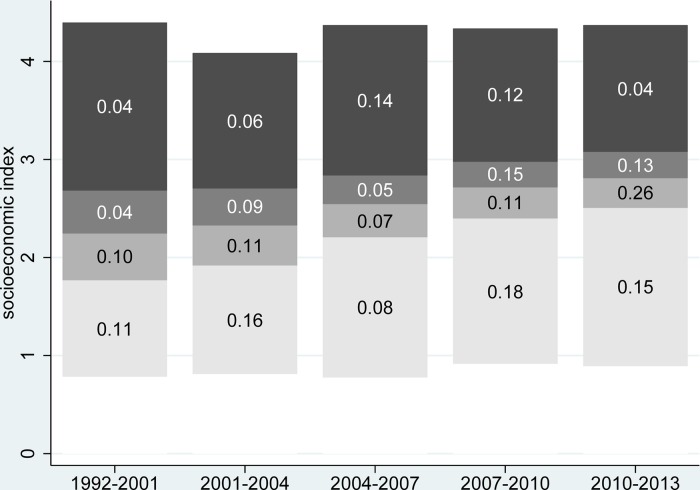
Quartiles of absolute socioeconomic status by time period. Numbers show malaria mortality rates for each socioeconomic quartile per 1000 person-years for 165 malaria deaths over 1.58 million person-years at the Agincourt HDSS, South Africa, from 1992 to 2012.

Household socioeconomic status increased significantly in absolute terms over the study period, with the median value increasing from 2.27 (95% CI 2.25–2.29) at the start to 2.77 (95% CI 2.76–2.78) by the end. Figure 4 shows quartiles of socioeconomic status over time with corresponding malaria mortality rates. The proportions of malaria deaths below the socioeconomic median level over the different periods were 69.8, 63.6, 42.1, 50.0 and 62.2%, respectively. Thus, at the start and end, malaria mortality was particularly associated with lower socioeconomic levels, but around the time when the visa requirement for travel to Mozambique was removed, malaria mortality became more widely distributed across socioeconomic status.

**Fig. 5.**
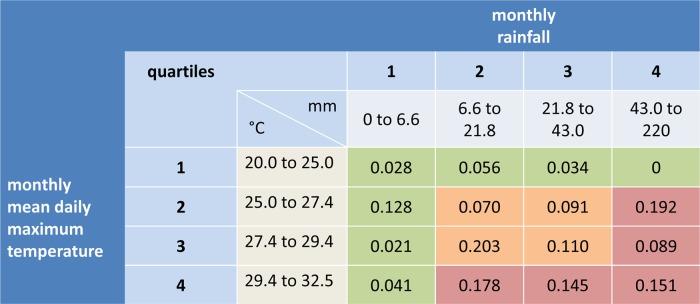
Monthly rainfall and monthly mean daily maximum temperature by quartiles in relation to malaria mortality in the following month, for 165 malaria deaths in 1.58 million person-years, over 252 months, expressed as rates per 1000 person-years. Green-shaded cells show rates where either or both rainfall and temperature were in the lowest quartile; orange-shaded cells show rates for the inter-quartile ranges of rainfall and temperature, and pink-shaded cells show rates where either rainfall or temperature were in the highest quartile, with neither in the lowest quartile.

In this sub-tropical climate, it is evident from Fig. 2 that yearly rainfall varied considerably and wetter years also had lower yearly mean daily maximum temperatures. There was much less variation in yearly mean daily minimum temperatures. On a monthly basis, there were considerably greater variations, reflecting seasonality, although these variations were more erratic for monthly rainfall and monthly mean daily maximum temperature than for monthly mean daily minimum temperature. Given the evidence of associations between rainfall and temperature, monthly rainfall and monthly mean daily maximum temperature were each classified into quartiles over the 252 months of observation. On the hypothesis that weather conditions in the preceding month are likely to determine malaria deaths by influencing mosquito breeding and parasite incubation, Fig. 5 shows the joint effects of monthly rainfall and temperature on malaria mortality rates in the month, following weather observations. Mean malaria mortality in the green-shaded cells, where either or both rainfall and temperature were in the lowest quartile, was 0.056 per 1000 person-years; in the orange-shaded cells corresponding to the interquartile ranges was 0.115 per 1000 person-years, and in the pink-shaded cells where either rainfall or temperature were in the highest quartile, with neither in the lowest quartile, was 0.142 per 1000 person-years.

Table 1 shows factors associated with malaria mortality on a bivariable basis, together with a series of Poisson multivariable regression models separately exploring basic characteristics, risk factors and weather parameters, plus a final overall model. In the ‘background’ model, both age group and time period were very significantly associated with malaria mortality. Age group was retained in all the models, but time period was dropped from subsequent models so as not to hide the effects of other covariates, which changed consistently over time. In the ‘risk factors’ model, higher socioeconomic status and being away for part of the year as a labour migrant were both significantly protective against malaria mortality. In the period after the visa requirement for travel to Mozambique was abolished, Agincourt residents who were not of Mozambican origin were significantly more likely to die of malaria. In the ‘weather’ model, high annual rainfall and high yearly mean maximum daily temperature as well as combinations of high monthly rainfall and high monthly mean daily temperature in the month before death were all significantly associated with malaria mortality. In the ‘overall’ model, most of the significant factors from the previous models maintained their associations with malaria mortality. The association between malaria mortality and yearly mean maximum daily temperature did not retain significance in this model.

**Table 1.** Malaria mortality rate ratios (MRR) from Poisson regression models of 165 malaria deaths by background, risk factors and weather, for 165 malaria deaths over 1.58 million person-years at the Agincourt HDSS, South Africa, from 1992 to 2012

Parameter	Level	Malaria deaths	Bivariate rate ratios		Background model^a^		Risk factor model^a^		Weather model^a^		Overall model^a^	
			MRR	95% CI	MRR	95% CI	MRR	95% CI	MRR	95% CI	MRR	95% CI
Sex	Female	81	Ref		Ref							
	Male	84	1.12	0.83–1.52	1.12	0.83–1.52						
Age group	Under 15	80	Ref		Ref		Ref		Ref		Ref	
	15–64	76	0.63*	0.46–0.86	0.60*	0.44–0.82	0.71*	0.51–0.98	0.61*	0.45–0.84	0.71*	0.51–0.98
	65 plus	9	0.97	0.49–1.93	0.94	0.47–1.86	0.93	0.47–1.85	0.95	0.48–1.88	0.93	0.47–1.85
Period	1992–1995	7	Ref		Ref							
	1995–1998	15	2.07	0.84–5.08	2.09	0.85–5.12						
	1998–2001	21	2.80*	1.19–6.58	2.85*	1.21–6.69						
	2001–2004	22	2.88*	1.23–6.75	2.97*	1.27–6.95						
	2004–2007	19	2.45*	1.03–5.83	2.56*	1.08–6.08						
	2007–2010	36	3.89*	1.73–8.74	4.08*	1.82–9.15						
	2010–2013	45	4.58*	2.06–10.2	4.83*	2.18–10.7						
Socioeconomic status	Q 1 – lowest	52	Ref				Ref				Ref	
	Q 2	49	0.92	0.62–1.36			0.90	0.61–1.32			0.90	0.61–1.33
	Q 3	33	0.61*	0.39–0.95			0.59*	0.38–0.92			0.60*	0.39–0.92
	Q 4 – highest	31	0.57*	0.36–0.89			0.54*	0.34–0.86			0.55*	0.35–0.87
Migration status	Permanent resident	158	Ref				Ref				Ref	
	Labour migrant	7	0.31*	0.15–0.67			0.34*	0.15–0.75			0.34*	0.15–0.75
Combination of visa requirement and Mozambican origin	Visa req; non-Moz	43	Ref				Ref				Ref	
	Visa req; Moz	29	1.20	0.73–1.98			0.97	0.58–1.60			0.97	0.59–1.60
	Visa free; non-Moz	24	2.14*	1.47–3.14			2.28*	1.55–3.36			2.28*	1.42–3.66
	Visa free; Moz	69	1.82*	1.14–2.92			1.60	0.99–2.60			1.61	0.93–2.79
Yearly rainfall	Q 1 – lowest	33	Ref						Ref		Ref	
	Q 2	54	1.61*	1.04–2.48					1.41	0.89–2.23	1.37	0.83–2.26
	Q 3	34	1.12	0.69–1.81					1.30	0.74–2.29	1.69	0.92–3.08
	Q 4 – highest	44	1.38	0.88–2.17					2.21*	1.29–3.79	1.98*	1.08–3.65
Yearly mean daily maximum temperature	Q 1 – lowest	26	Ref						Ref		Ref	
	Q 2	39	1.39	0.85–2.28					1.60	0.91–2.53	0.98	0.55–1.73
	Q 3	51	1.73*	1.08–2.77					2.26*	1.29–3.96	1.38	0.70–2.74
	Q 4 – highest	49	1.63*	1.01–2.62					2.43*	1.33–4.45	1.66	0.83–3.32
Preceding month's temperature and rainfall^b^	One/both in Q1	32	Ref						Ref		Ref	
	Inter-quartile ranges	39	2.08*	1.30–3.32					2.00*	1.25–3.21	2.21*	1.38–3.53
	One/both in Q4 and neither in Q1	94	2.58*	1.73–3.85					2.53*	1.70–3.77	2.60*	1.75–3.87

*Rate ratio significantly different from unity (*p* < 0.05).

^a^Each multivariable model is adjusted for all of the factors for which values are shown.

^b^The three levels of this parameter correspond to the green, orange and pink shaded cells in Fig. 5, respectively.

## Discussion

Clearly malaria mortality as measured at the Agincourt HDSS increased over the 21-year period of observation, with malaria-specific mortality rates ranging from 0.02 to 0.2 per 1000 person-years, corresponding to recent increases for malaria in South Africa as reported by the WHO [3]. Epidemiological factors that may have been associated with this increase are of great public health importance in an era of potential malaria elimination [4]. Regional programmes such as LSDI and MOSASWA may have influenced cross-border and autochthonous malaria transmission in the area [12]. Local epidemiological factors were amenable to exploration using the detailed Agincourt HDSS cohort data, even though a cohort design of this kind cannot elucidate cause and effect.

### Individual and sociopolitical factors

South Africa has undergone rapid sociopolitical development in the last two decades, some aspects of which are likely to have influenced malaria transmission. This has led to changes in patterns of labour migration [27] which inevitably have consequences for malaria control [28]. While malaria mortality remained associated with relative poverty [29], evidence of changing malaria mortality by socioeconomic quartiles in Fig. 4 may reflect associations between resources and mobility after visa restrictions were eased between South Africa and Mozambique in 2005. Although we had no data on individual travel to Mozambique as a putative cause, higher malaria mortality was observed after visa requirements were lifted. Equally, increased employment opportunities in nearby game lodges within South Africa's endemic region may have led to increased autochthonous cases among those with salaries. In the opposite sense, labour migrants from the endemic area, many of whom work in the mining and other commercial sectors in non-endemic areas of South Africa, were considerably protected from malaria mortality. Malaria deaths occurred in all age groups, which is consistent with low levels of immunity in the population, though malaria mortality rates were significantly lower in the 15–64-year age group. Conversely adults are possibly also more likely than children to travel, leading to greater exposure and acquisition of infection elsewhere. There was no evidence in these data that the considerable HIV/AIDS mortality epidemic, previously documented to have affected the Agincourt population and peaking around 2006–2007 [30], gave rise to any corresponding peak in malaria mortality, despite affecting other infectious causes of death. There has however been a suggestion from Mozambique that HIV co-infection can increase malaria severity and mortality [31], and anti-retroviral therapy may affect the efficacy of lumefantrine for malaria treatment unless an extended course is given [32]. These factors may have contributed to recent increases in Agincourt malaria mortality, given the relatively large proportion of the adult population living with HIV. A population-based HIV prevalence study in 2010–2011 found that 19.4% of adults (15 years and over) were living with HIV [33].

### Weather and climate change factors

Both short-term weather and longer-term climate change factors are issues of concern for malaria control. The sub-tropical climate of the Agincourt area means that malaria transmission is regulated both by rainfall and temperature over various seasons, which was the rationale for the combined presentation of these factors in Fig. 5. Generally there was lower malaria mortality in months following dry and/or cold months, and weather emerged as a major overall determinant of malaria mortality. Equally, it has to be recognised that malaria infections imported from outside the area will not necessarily have been subject to the same meteorological factors.

However, there was also a steady overall trend towards higher temperatures over the 21-year period, and if this continues it could contribute to significant future increases in malaria. From these data, it is not possible to say unambiguously that the overall temperature trend was responsible for increasing malaria mortality, though it may have been a contributory factor. Some models predict that climate change could considerably increase malaria transmission potential in southern Africa in coming decades [34]. It is also possible that low malaria transmission patterns in relatively non-immune populations are particularly sensitive to variations in weather [35].

### Methodological strengths and weaknesses

Compared with many studies of malaria mortality, the methods used here have distinct strengths and weaknesses. This longitudinal dataset derived from the application of standard methods over a 21-year period within a defined population area offers a unique opportunity to track developments in malaria mortality. All deaths in the Agincourt HDSS population are routinely followed up and causes of death attributed using standard VA techniques, so there is no bias arising from deliberately seeking out malaria deaths. Similarly there is no health-seeking bias involved, unlike data originating from passive case detection at health facilities, which may underestimate asymptomatic cases or miss cases seeking testing and treatment at private pharmacies. This is important in a low-transmission setting like Agincourt where individual immunity to malaria is generally low and malaria infections can rapidly lead to fatal consequences, possibly before treatment is sought. Anecdotally from the VA narratives, a proportion of cases did not seek any treatment. The weakness of the VA approach for documenting malaria deaths is that there can be no supporting parasitological or other biomedical evidence, and hence it is not possible to derive parasite rates or case-fatality rates. Although the InterVA-4 model has been shown across a wide range of settings to deliver evidence of malaria mortality consistent with findings from the Malaria Atlas Project, WHO and the Global Burden of Disease Study [15, 16], VA is usually not regarded as the method of choice for documenting malaria mortality. However, the malaria mortality rate of 0.10 per 1000 measured here for Agincourt, on the borders between Bohlabela District to the north (0.02 per 1000) [8] and Bushbuckridge Municipality to the south (0.36 per 1000) [9] is very plausible. Since it is unlikely that a consistent and systematic clinical population-based follow-up specifically for malaria deaths could have been justified over a 21-year period in a setting where malaria is a relatively rare disease, applying consistent VA surveillance methods to data spanning a generation was a considerable strength of this study, enabling robust observations of malaria mortality trends in this population. It was however impossible to make any systematic assessment of whether individual malaria deaths were associated with autochthonous or travel-related transmission, though some cases were anecdotally reported not to have travelled outside South Africa. In the absence of data in this population on malaria infection rates, it is also possible that the observed increase in malaria mortality over time reflects an overall reduction in infection and consequently in acquired immunity, making the disease increasingly dangerous.

### Implications for malaria elimination programmes in South Africa and elsewhere

The WHO manual for malaria elimination [36] does not set specific epidemiological criteria differentiating between malaria control and elimination settings, and the epidemiological quantification of declining malaria is complex [37]. However, our findings here suggest that South Africa is still some way from achieving elimination in the relatively small areas of the country continuing to experience transmission. Although there was reasonable optimism in 2012 [38], recent developments, reflected both in these results and the 2015 WHO World Malaria Report [3], suggest that there is still some way to go.

Our findings that malaria mortality has increased over two decades, in an area that might have contemplated embarking on elimination activities in the early 1990s, are a matter for concern, notwithstanding sociopolitical and climatic factors that may have contributed to the changes [39]. For other African settings, as countries increasingly achieve control and contemplate elimination, it is likely that the road to elimination will not be smooth. If pre-elimination and elimination phases span decades, earlier population immunity will dwindle and case-fatality may increase, among other factors. Understanding the prospects for elimination therefore requires a long-term view of local malaria epidemiology [40].

Countries sharing borders with areas of higher transmission, as seen in this study, present special problems. Modelling suggests that malaria interventions at borders are likely to be necessary [41]. Although local flights from Mozambique to South Africa may well undergo routine disinsection as provided for in the WHO International Health Regulations [42], the same procedures are not routinely applied to ground transportation, which may comprise the bulk of traffic, and potentially convey infective vectors. Effective trans-national collaboration is likely to be a key to success [43].

## Conclusions

Malaria, even in the low transmission setting of Agincourt, has persisted as a seemingly intractable public health problem over the last two decades and mortality shows no sign of decreasing. Having consistent individual HDSS data collected over more than two decades was essential for undertaking these analyses. A number of sociopolitical factors as well as weather and longer-term climate appear to be associated with malaria mortality levels, implying that there can be no single strategy for achieving better control and possible elimination. These findings will be important for other settings, which manage to reduce malaria cases and deaths to low levels, but then encounter difficulties in making further progress towards eliminating transmission. It is clear that political, economic and climatic considerations will have to be successfully mitigated and managed during the coming years, alongside the provision of excellent health services, if the global Sustainable Development Target of eliminating malaria by 2030 is to be met.

## Data Availability

Population-based data from the Agincourt HDSS are routinely archived for public access in the INDEPTH data repository, http://www.indepth-ishare.org/index.php/home
